# *Shigella sonnei* Outbreak among Homosexual Men, London

**DOI:** 10.3201/eid1209.060282

**Published:** 2006-09

**Authors:** Oliver Morgan, Paul Crook, Tom Cheasty, Brian Jiggle, Isabelle Giraudon, Harriett Hughes, Stephen-Morris Jones, Helen Maguire

**Affiliations:** * Health Protection Agency, Centre for Infections, London, United Kingdom;; † South West London Health Protection Unit, London, United Kingdom;; ‡ Health Protection Agency London Region, London, United Kingdom;; § European Programme for Intervention Epidemiology Training, London, United Kingdom;; ¶ University College London Hospital, London, United Kingdom;; # St Georges Hospital Medical School, London, United Kingdom

**Keywords:** Shigella sonnei, HIV, homosexuality, disease outbreaks, letter

**To the Editor:** In the summer of 2004, genitourinary medicine clinics in London reported cases of *Shigella sonnei* with a novel phage type pattern (later designated PTQ). Outbreak case finding involved local laboratories and genitourinary medicine physicians in London, as well as the national reference laboratory. A case was considered confirmed if *S. sonnei* PTQ was isolated from January 2004 through April 2005, and the patient had not traveled outside the country the week before illness. Possible cases were defined as for confirmed cases but were so designated when patient had a history of foreign travel in the week before illness or when travel history was unknown. From October 2004, when we became aware of the outbreak, until December 2004, we conducted telephone interviews with newly identified case-patients. For cases that occurred before October 2004, and from January 2005 through April 2005, information was obtained from laboratory records only.

Strains were phage typed by using the scheme described by Hammerstrom, Kallings, and Sjoberg, according to a protocol supplied by R. Wollin (*1**,**2*). The scheme consists of 11 phages and is based on the typing of the rough phase II variant of *S. sonnei*. The scheme comprises defined phage types (PT) 1–100 and provisional PTs A–P. Cultures were grown overnight on MacConkey agar, and a rough colony was placed in nutrient broth and grown for 18 hours at 37°C. The broth culture was then used to flood a nutrient agar plate and, once dry, spotted with the 11 phages and incubated at 37°C for 5 hours. The patterns of lysis were recorded and compared with those indicated on the typing chart. All isolates were screened for resistance to a panel of antimicrobial agents by an agar incorporation method with Iso-Sensitest agar (Oxoid, Basingstoke, UK).

We identified 16 confirmed and 54 possible cases. Specimens from all 70 patients had the same unique pattern of lysis when phage typed, had the same profile when examined by pulsed-field gel electrophoresis, and were resistant to ampicillin, streptomycin, spectinomycin, sulfonamides, tetracyclines, and trimethoprim.

Cases occurred at a low frequency during the first half of 2004, followed by a large increase in August, September, and October (Figure). All case-patients (N = 48) were men, mean age 37 years (range 18–58 years). Five persons designated possible case-patients had traveled abroad in the week before illness (United States, France, Vietnam, Turkey, and 1 unknown destination). Of patients for whom HIV status information was available, nearly all were HIV positive (n = 30/32).

**Figure:**
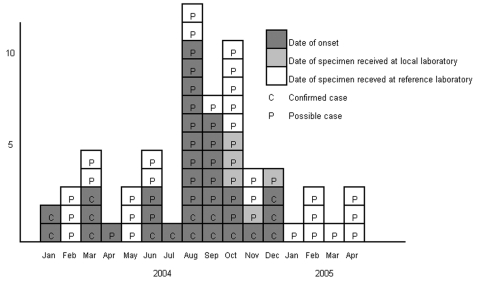
Confirmed and possible cases of *Shigella sonnei* PTQ by earliest recorded date, London, January 2004–April 2005.

From October 2004 through December 2004, we identified 20 case-patients and interviewed 17 (85%). All were men who had sex with men (MSM). Reported symptoms were diarrhea (n = 15), abdominal pain (n = 14), fever (n = 10), blood in stools (n = 7), and vomiting (n = 6). In the week before illness, 15 reported sex with another man, about half with a casual partner, and mostly with 1 (9/15) or 2 (3/15) different men. No common sex venue was identified. Most (12/15) reported participation in oral and anal sex, and 6 reported oral-anal contact. Three patients recalled that their partner had had diarrhea around the time of sexual intercourse. Of 7 respondents who were asked, 3 reported using a condom during anal intercourse, and none reported using any barrier during oral intercourse.

That all cases were men, and many were HIV-positive MSM, who reported having sex the previous week, strongly suggests that male homosexual sex was the mode of transmission. The shape and timeframe of the epidemic curve indicates person-to-person transmission and rules out foodborne transmission linked to a gay venue. The predominance of HIV-positive homosexual men in the outbreak may be due to more symptomatic disease (from compromised cell-mediated immunity or achlorhydria [3]), more unprotected sex with other HIV-positive men (*4*), and greater likelihood of seeking healthcare.

Sexual transmission of shigellosis between MSM was first reported in the United States during the 1970s (*5*), and recent outbreaks have been reported in San Francisco (*6*), Canada (*7*), Australia (*8*), and Germany (*9*). The London outbreak coincided with an outbreak of *S. sonnei* in Berlin, Germany (*10*). Of the 17 Berlin case-patients, 14 were MSM. Isolates from 10 Berlin patients were subsequently tested by the same reference laboratory in London and confirmed to also be PTQ, which suggests a link between these 2 outbreaks, even though none of the London interviewees reported travel to Berlin.

Although the earliest identified case occurred in January 2004, *S. sonnei* PTQ may have been circulating among the MSM community for a longer period. The discovery of an outbreak of a novel phage type underlines the importance of prompt strain-typing for public health investigations and the benefit of good links between local clinicians, laboratories, and public health professionals. Additionally, local gay media and voluntary organizations were valuable partners for disseminating preventative health messages across London when the outbreak was in the early stages. This outbreak raises the possibility that the mobility and increased high-risk sexual practices among MSM in Europe (*4*) might facilitate mixing between sexual networks, thus causing potential for international outbreaks of sexually transmitted infection.
